# Advancing Precision Oncology with Digital and Virtual Twins: A Scoping Review

**DOI:** 10.3390/cancers16223817

**Published:** 2024-11-13

**Authors:** Sebastian Aurelian Ștefănigă, Ariana Anamaria Cordoș, Todor Ivascu, Catalin Vladut Ionut Feier, Călin Muntean, Ciprian Viorel Stupinean, Tudor Călinici, Maria Aluaș, Sorana D. Bolboacă

**Affiliations:** 1 Department of Computer Science, West University of Timișoara, Vasile Pârvan Blvd., No. 4, 300223 Timișoara, Romania; sebastian.stefaniga@e-uvt.ro (S.A.Ș.); todor.ivascu@e-uvt.ro (T.I.); 2 Department of Surgery-Practical Abilities, “Iuliu Hațieganu” University of Medicine and Pharmacy, Marinescu Street, No. 23, 400337 Cluj-Napoca, Romania; 3 Department of Public Health, Faculty of Political, Administrative and Communication Sciences, Babeș-Bolyai University, 400084 Cluj-Napoca, Romania; 4 First Discipline of Surgery, Department X-Surgery, “Victor Babeș” University of Medicine and Pharmacy, E. Murgu Sq., No. 2, 300041 Timișoara, Romania; catalin.feier@umft.ro; 5 Medical Informatics and Biostatistics, Department III-Functional Sciences, “Victor Babeș” University of Medicine and Pharmacy, E. Murgu Sq., No. 2, 300041 Timișoara, Romania; cmuntean@umft.ro; 6 Department of Computer Science, Babeș-Bolyai University, M. Kogalniceanu Str., No. 1, 400084 Cluj-Napoca, Romania; ciprian.stupinean@gmail.com; 7 Department of Medical Informatics, “Iuliu Hațieganu” University of Medicine and Pharmacy Cluj-Napoca, Louis Pasteur Str., No. 6, 400349 Cluj-Napoca, Romania; tcalinici@umfcluj.ro; 8 Department of Oral Health, “Iuliu Hațieganu” University of Medicine and Pharmacy Cluj-Napoca, Victor Babeș Str., No. 15, 400012 Cluj-Napoca, Romania; maria.aluas@umfcluj.ro; 9 Center for Bioethics, Babeș-Bolyai University, Avram Iancu Str., No. 68, 400083 Cluj-Napoca, Romania

**Keywords:** digital twins in healthcare (DTH), virtual twins in healthcare (VTH), oncology, patient-centered care

## Abstract

Digital twins (DTHs) and virtual twins (VTHs) in healthcare represent emerging technologies towards precision medicine, providing opportunities for patient-centric healthcare. Our scoping review aimed to map the current DTH and VTH technologies in oncology, summarize their technical solutions, and assess their credibility. A systematic search was conducted in the main bibliographic databases, identifying 441 records, of which 30 were included. The studies covered a wide range of cancers, including breast, lung, colorectal, and gastrointestinal malignancies, with DTH and VTH applications focusing on diagnosis, therapy, and monitoring. The results revealed heterogeneity in targeted topics, technical approaches, and outcomes. Most twining solutions use synthetic or limited real-world data, raising concerns regarding their reliability. Few studies have integrated real-time data and machine learning for predictive modeling. Technical challenges include data integration, scalability, and ethical considerations, such as data privacy and security. Moreover, the evidence lacks sufficient clinical validation, with only partial credibility in most cases. Our findings underscore the need for multidisciplinary collaboration among end-users and developers to address the technical and ethical challenges of DTH and VTH systems. Although promising for the future of personalized oncology, substantial steps are required to move beyond experimental frameworks and to achieve clinical implementation.

## Simple Summary

Our scoping review highlights the advancements in digital (DTHs) and virtual (VTHs) twins in healthcare for diagnosing, treating, and monitoring cancer patients, specifically those with breast, lung, or gastrointestinal cancers, while also addressing pain management. The proposed twining solutions lack fairness and credibility, highlighting the need for the involvement of researchers with diverse expertise. The reported findings show that DTHs and VTHs are advantageous in precision healthcare, providing a comprehensive perspective on the advancements in precision medicine for cancer management. Nevertheless, there is a considerable journey ahead before they can be effectively used in daily clinical settings.

## 1. Introduction

A digital twin (DT) refers to a digital replica of a physical entity, whether living or non-living, designed to mirror the real world through detailed information about the entity that it represents, sometimes enabling real-time monitoring, analysis, and simulation [1,2,3]. Virtual representation interconnects with its physical counterpart, enabling real-time data exchange and interaction [3]. A core feature of DTs is their capacity to synchronize with their physical entities, enabling bidirectional communication and continuous updates. Virtual twins (VT) extend the DT concept by comprehensive simulation capability, with continuous improvement through real-world data feedback as the key feature [1]. The penetration of DT and VT in healthcare is slow [4,5] compared with other domains, such as the aerospace industry [6,7], manufacturing [8,9,10], and urban planning [11,12,13]. 

In medicine, a digital twin model incorporates a human being as a physical entity and establishes a connection between the digital representation of the human in a virtual world and a digital model that captures the features of the physical object. A digital thread connects virtual representations and digital models to ensure data sharing [14,15]. The focus of digital twins is on real-time monitoring and prediction. Table 1 summarizes the main architecture and the characteristics of the three-layer DT architecture [16,17,18]. However, different research teams have proposed different numbers of layers with different or similar functions (e.g., three-layer architecture with a data acquisition layer [19] or data exchange layer [20], four- [21], five- [22,23], or six-layer architecture [24,25]).

Virtual twins (VTs) are tools that are used to simulate *ad infinitum* hypothetical scenarios and outcomes based on varying inputs. Virtual twins provide active user engagement, offering the possibility of adjusting parameters, changing conditions, or direct interaction with the virtual representation. The key technical features of a VT solution are simulation and user engagement (e.g., 3D modeling) and integration with planning (e.g., testing and simulation to ensure model perfection and continuous improvement). To accelerate collaboration, VTs face the key challenge of integrating end-to-end systems. Table 2 presents the key differences between DTs and VTs [26,27,28,29].

**Figure 1:**
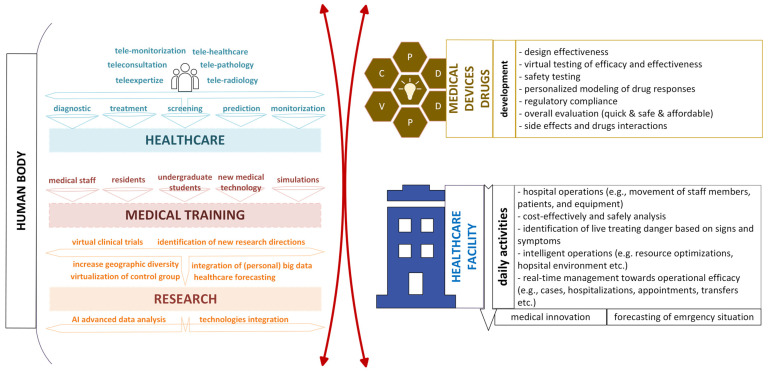
The potential of medical features of digital and virtual twins for health. PDDPVC (medical devices) refers to Project–Design–Development–Production–Validation–Commercialization.

To support personalized medicine and better health, consortiums have been developed at national level (e.g., Swedish Digital Twin Consortium (SDTC) [30]), European level (e.g., DigiTwin Consortium [31]; European Virtual Human Twins Initiative [32], EDITH—European Virtual Human Twin [33]), and global level (such as the Digital Twin Consortium, DTC^®^ [34]). Generally, DT and VT consortiums look for solutions in multiple domains, including health, life sciences, and pharmacy. Digital and virtual twins for healthcare (DTHs and VTHs) are expected to revolutionize the entire healthcare system. For example, *in silico* environments for health can find their main applicability in medicine in the following three domains: the human body, medical devices, and healthcare facilities [35,36,37,38,39,40] (Figure 1). Twinning technical solutions can support personalized advanced telemedicine applications with real-time representation and integration of patient data and health status, enabling in-place interventions when needed. Medical training can be gained from twinning solutions by simulating real-world scenarios for any level of education (e.g., undergraduate students, residents, continuing medical education, and patient education). Digital twins of the human body enable immersive simulations for the comprehension of complex cases, while virtual clinical trials provide a platform to see the outcomes of different scenarios in a virtual environment. The potential of digital and virtual twins in research is derived from AI analytics, forecasting, prediction, virtual clinical trials, and so on. Regarding medical devices and drugs, DTHs and VTHs can assist researchers in the development phase by enabling real-time modeling and testing, in the phase of device or drug prototyping, preclinical evaluation for safety and monitoring the side effects or drug interactions, etc. At the healthcare facility level, twinning solutions can be used to optimize daily operations through operational simulations for better forecasting, resource management and allocation, prediction of equipment maintenance needs, and the improvement of patient flow [35,36,37,38,39,40].

Researchers have previously reported DTH and VTH solutions for oncology, highlighting their contribution to cancer prediction [41]. Sager [42] introduced the concept of DTHs in oncology, emphasizing their possible roles in biomarker monitoring, optimization of treatment, patient monitoring, training of medical staff, and optimization of clinical studies. Overviews and reviews of the DTHs and VTHs state-of-the-art in oncology are available in the scientific literature; however, these studies emphasized the progress in one topic (e.g., pharmacology in immuno-oncology [43], medical imaging in clinical oncology [44], treatment of endometrial cancer [45], roadmap towards cancer personalized theragnostic application [46], or reported more than oncology (e.g., cardiovascular diseases and cancer [47]. Systematic reviews of DTH and VTH solutions for oncology are scarce in the scientific literature. Shen et al. [48] investigated the effectiveness of digital twins and reported 12 articles, 4 of which included patients with cancer [49,50,51,52].

Despite the growing interest in DTH and VTH solutions across the globe, the limited available solutions have not been systematically summarized in terms of technical and clinical features. Systematic, validated, and patient-centered DTH and/or VTH research is needed to better understand their potential and limitations in oncology. The aim of our study was threefold, as follows: to map DTHs or VTHs that address the needs of patients with cancer, to summarize the proposed technical solutions, and to assess the credibility of the available technologies.

## 2. Materials and Methods

Preferred Reporting Items for Systematic Reviews and Meta-Analyses for Scoping Reviews (PRISMA-ScR) was used in this study [53].

In our study, we distinguish between digital twins in healthcare (DTH) and virtual twins in healthcare (VTH). Digital twins in healthcare feed the digital replica in real-time, periodically, or at least once during the lifecycle. This allows for dynamic interaction between the individual and its digital counterpart. Virtual twins in healthcare (DTHs) are primarily used for simulations, where data flows in one direction, from the patient to the virtual model, enabling the exploration of potential scenarios with no continuous real-time updates.

### 2.1. Eligibility Criteria

Papers reporting the effectiveness of DTHs or VTHs in patients with any type of malignant tumor, regardless of whether they addressed diagnostic, therapeutic, or prognostic factors, were eligible to be mapped. Scientific articles that met the following criteria were included in the assessment: manuscripts published before 8 August 2024; written in English; involved human participants, data, or synthetic data generated based on real data; and presented the effectiveness of DTHs or VTHs for cancer. Regardless of the applied design, articles were included if they reported an original study, even at the pilot or feasibility level. Scientific articles were excluded when they did not fit into the conceptual framework of the study, did not include a measure of the effectiveness of DTHs or VTHs, or did not include any evidence regarding the input of real data or applicability to patients with cancer. Reviews, overviews, and systematic reviews were excluded if they did not describe at least a presentation of a case study. Reviews, overviews, and systematic reviews were used to identify eligible manuscripts.

### 2.2. Information Source, Search Strategy, and Timing

The search of bibliographic databases was performed from 8 to 15 August 2024. Table 3 includes the bibliographic databases used in this process, a summary of the search string, and the filters applied per database. The search strategies were initially created by one of the authors and then enhanced through team discussion. The Web of Science (WoS) was used in “All Databases” to capture all possible eligible items.

The search was expanded by searching DTH or VTH research project websites to identify pertinent documents, if available. The websites listed in Table 4 were used on 19 August 2024.

The search results were exported to a Microsoft Excel spreadsheet and duplicates were identified using the Duplicate Values function in Conditional Formatting.

### 2.3. Article Selection Process and Data Charting

Two reviewers performed the title-abstract selection process and individually classified each item as follows: 0 = include, 1 = not DTH or VTH, 2 = not cancer, 3 = no original article, 4 = fully theoretical, 5 = conference abstract, and 6 = not humans. Items that passed the initial screening and were classified as 0 were included in the full-text screening. Three reviewers screened the full papers and classified the items as follows: 0 = include or 9 = exclude. We included the full paper in the evaluation whenever a study was reported both as a conference proceeding and as a full paper. Consensus resolved disagreements regarding the selection of the studies.

Data-charting followed the current recommendations published by Pollock et al. [54]. Two standardized in-home data-charting forms were used, as follows: one to capture the health-related characteristics of the eligible studies and another to collect the technical features of the DTHs or VTHs. The data-charting of the study characteristics included the following:

- Country where the authors were affiliated;
- The study type;
- Disease;
- Patient characteristics (e.g., number of patients, sex, and age) and setting (e.g., healthcare facility/virtual facility);
- Twin type (e.g., one body system/one body organ/body function/finer body component levels (cellular, subcellular)/entire human body/other);
- Intervention (e.g., diagnostic/therapy/prognostic/monitoring/other);
- Outcome(s);
- Reported results.

The technical team charted the following data:

- Hardware;
- Middleware;
- Software;
- Key technologies;
- Data flow (unidirectional/bidirectional);
- Analytical methods (AI/ML/decision algorithm/other-specify);
- Fairness (algorithm fairness—minimizing existing biases or inequalities);
- Model performances;
- Credibility (no/partial/complete evidence/sufficient credibility/certified credibility). Certified credibility means certified by a regulatory agency;
- Computational resources (HPC/cloud/edge computing/distributed);
- Privacy;
- Confidentiality.

The presentation and/or discussion of ethical issues and misuse of the source documents were also reviewed and summarized.

Eight reviewers evaluated the full-text, four extracted health-related data, and another four extracted technical information. The reviewers individually mapped the data. An impartial moderator oversaw the reviewers’ discussions of the disagreements and reported the agreed-upon information.

PRISMA 2020 [55] was used to report the flow from the search to the inclusion of articles. The reported results were summarized using narrative synthesis.

The manuscripts were also evaluated to assess compliance with the IMRaD standard for publishing in medical journals [56].

## 3. Results

### 3.1. Source of Evidence

**Figure 2:**
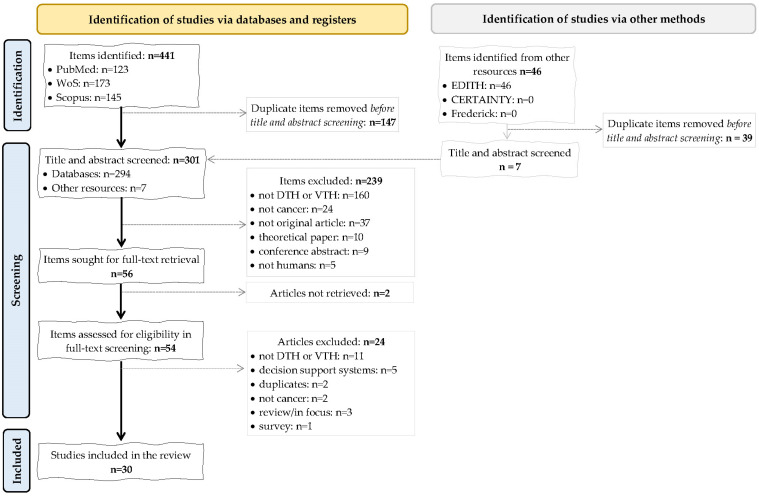
Flow from manuscript identification to inclusion. WoS—Web of Science, DTH—digital twin in healthcare, VTH—virtual twin health in healthcare.

The search retrieved 441 items, 147 duplicates were removed, and 56 passed the title-abstract screening. We could not retrieve 2 full texts, and 30 of the 54 articles were summarized (Figure 2).

Papers published from 2020 to date—with most of them published in 2023 (14 papers), nine published in 2024, five in 2022, and the majority were journal articles with research conducted in the USA, as per author affiliation—were included in the analysis (Table 5).

### 3.2. Synthesis of the Individual Sources of Evidence

#### 3.2.1. General Characteristics and Targeted Clinical Applicability

The researchers carried out all evaluated studies in laboratory settings, focusing on most of the cases in one body organ, frequently targeting therapy or diagnosis (Table 6).

The researchers did not always report the source of the data and frequently used previously reported data. Jamshidi et al. [72] and Moztarzadeh et al. [78] used data collected in Portugal. Chang et al. [79] used 1372 CBCT (cone-beam computer tomography) image sets as their source of data. Information about patients in the evaluated papers, whenever available, is scarce, with only a limited number of articles reporting the patients’ sex and age. Ahmadian et al. [60] used data from one female patient of 51 years with lung cancer and computed tomography (CT)/magnetic resonance imaging (MRI). Lin et al. [62] developed algorithms based on fecal immunochemical test for colorectal cancer screening data from 5,417,699 residents aged 50–69 years. The targeted patients included by Bahrami et al. were aged from 40 to 68 years [66] and, respectively, 67.6 ± 13.4 years (range 43 to 85 years) [66]. The patients described by Tai et al. [71] were age between 40 and 70 years, and 35.7% were women. Kim et al. [73] targeted male patients with a mean age of 67.37 years (range: 46 to 83 years). The average age and standard deviation of patients reported by Peterson et al. [77] was 53.4 (10.2) years.

Real-world data were used to construct DTHs or VTHs or to generate virtual cohorts. Bahrami et al. [50] generated 3000 virtual patients based on information retrieved from 20 patients (10 women and 10 men) aged 40 to 68 years. The DTH developed by Yankeelov et al. [63] is based on data retrieved from a female patient. Bahrami et al. [66] generated 500 virtual patients based on real data from 8 patients. Meng et al. [74] simulated a metagenomic dataset containing 10,000 samples based on real data, with each sample defined by 1000 features. Sharma et al. [80] used 1013 Pap smear images (224 × 224 × 3) and 4103 cells to test the performance of the proposed algorithm. Qi and Cao [61] generated a virtual cohort of 1000 patients with 4109 lesions. Chaudhuri et al. [49] referred to only 100 *in silico* cohorts in their paper.

The evaluated twinning solutions exhibited considerable variation in focus, methodologies, and reported outcomes (Table 7). Some solutions are designed for diagnostic purposes, whereas others prioritize therapy or monitoring.

#### 3.2.2. Technical Evaluation of the Proposed Solutions

Most studies acknowledge the use of HPC computing [59,60,68,69,70,74,77,79,81,82,83], a smaller number mention cloud computing [49,64,71,73,75,76,78], and only one mentions edge computing [80]. Imaging devices such as MRI [49,69,77,81] or CT [77] and IoT devices [57,58,65,71,78,80] have also been used to construct DTHs and VTHs. Proton beam machines [79] and T-cell-tracking devices [82] have been reported as hardware supports. All of the reported twining solutions present a software layer (Table 8).

Confidentiality and privacy are acknowledged in only a few articles. Zhang et al. [57] ensured privacy and confidentiality through secure data handling and vulnerability detection. Meraghni et al. [58] acknowledged privacy and confidentiality as challenges and highlighted the need to address these issues in future works. Lin et al. [62] used de-identified data from national registries and population-based screening programs, implying that privacy and confidentiality were considered. Bahrami et al. [50] did not explicitly present privacy and confidentiality; however, the use of de-identified data suggests that these considerations were addressed in the design of the study. Gamage et al. [64] acknowledged privacy and confidentiality as critical aspects of the platform. Bahrami et al. [66] acknowledged privacy and confidentiality, as their study adhered to ethical standards and used anonymized data to protect the patients.

In general, fairness was not explicitly addressed in the evaluated manuscript. Meraghni et al. [58] used customization of the DTH for each patient’s unique physiology as a support for fairness. Fairness is sometimes addressed through (a) the personalization of treatment protocols, ensuring that each patient receives a tailored approach based on their specific characteristics (e.g., Yankeelov et al. [63], Bahrami et al. [66], and Bahrami et al. [67]); and (b) the platform’s ability to personalize treatments and diagnostics for individual patients, potentially reducing disparities in healthcare outcomes by tailoring interventions to each patient’s unique physiological profile (Gamage et al. [64]).

The data flow was bidirectional in 22 out of the 30 evaluated manuscripts (Table 9). Credibility was partially supported in 16 out of 30 cases, while sufficient credibility was reported in 9 out of the 30 manuscripts (Table 9).

#### 3.2.3. Appraisal of Source of Evidence

Eight of the papers evaluated described the contents of the sections [49,57,58,59,60,73,78,80]. Twelve articles did not respect the IMRAD structure [50,51,57,58,59,60,62,65,66,67,72,76], and eight did not present their methods in sufficient detail to allow replication/reproduction [50,57,62,71,72,73,74,78].

Good practice guidelines in scientific writing were not respected by most of the evaluated papers [50,57,62,71,72,73,74,78]. The information related to the methods was provided in the Results or Discussion Sections, the authors cited other researchers work in the Results Section, the study results were listed in the Introduction Section, the metrics of performance were revealed in the Methods Section but were not reported in the Results Section, there was reference to a section that does not exist in the manuscript, and there were conclusions that were not supported by the reported results. Chaudhuri et al. [49] reported their results in the Introduction Section. Christenson et al. [81] referred to “Section 3.5”, however, this section does not exist in the manuscript. The self-explanatory rule of reported figures was not always respected (e.g., Yankeelov et al. [63]), and sometimes the reported figures were not sufficiently clear (e.g., Jamshidi et al. [72]).

The limitations of the proposed approach were discussed in less than half of the evaluated manuscripts [49,51,61,62,69,72,75,77,78,80,81,82,83].

A discussion of ethical implications was highlighted in only two papers. Kolokotroni et al. [83] highlighted privacy, security, integrity, and patient consent. Moztarzadeh et al. [78] emphasized the importance of data protection and compliance with regulations (e.g., the General Data Protection Regulation) in the development of medical digital twin platforms.

## 4. Discussion

In our scoping review, we emphasize the developments of DTHs and VTHs to aid in the diagnosis, therapy, and monitoring of patients with malignancies, particularly in breast, lung, or gastrointestinal cancers, as well as in pain management. The reported results highlight a paucity of fairness and sufficient credibility of the proposed twining solutions, showing the need for multidisciplinary involvement of researchers with different levels of expertise. Our scoping review provides evidence for precision medicine targeting malignancies and supports the utility of DTHs and VTHs in precision healthcare.

### 4.1. Current Research and Developments

The penetration of digital twin concepts in medicine came later than it did in other domains, considering its earliest introduction (“living model” born in the 1960s by NASA as a simulation environment for the preparation for the Apollo mission [84]). Digital twin and virtual twin concepts fit perfectly into “there is no disease, there is a patient” idea and support precision and personalized medicine [85,86] for the “next generation of evidence-based medicine” [87].

The analyzed DTH and VTH solutions generally address specific problems, and some, but not all (e.g., thermography), arise from the needs of clinicians to find current-practice solutions. Diagnostics or therapy are the most common topics seen in the evaluated manuscript targeting the most prevalent cancers, namely breast, lung, colon, and rectum cancer in women; and prostate, lung, colon, and rectum cancer in men [88]. If we read between the lines, we can find the added value of experts from different disciplines, but it is not sufficiently presented in the manuscripts. It is frequently unclear whether and to what extent end-users are involved at any level in the development of the proposed solutions.

#### 4.1.1. Breast Cancer

Meraghni et al. [58] used thermography, a radiation-free method, as a non-invasive technique for breast cancer detection. They used physical space, data processing, and decision-making layers to show that the temperature differences between healthy tissues and tumor-affected tissues vary based on individual anatomy and environmental factors. However, to increase the accuracy of these methods, a new source of data must be incorporated. The clinical dimension of such a solution for daily practice is unclear, considering that the accuracy of thermography in diagnosis of breast cancer is low (69.7% [89]).

Yankeelov et al. [63] combined biology-based and data-driven modeling and simulated patient-specific responses to breast cancer therapies towards optimized treatment protocols on an individual basis. The reported results show the individual benefits of drug concentration and tumor volume when the optimized treatment protocols are used (Table 7).

Gamage et al. [64] introduced the 12 LABOURS Digital Twin Platform and showed how an automated breast biomechanics workflow could assist clinicians in diagnosing and treating breast cancer. However, the openness and willingness of clinicians towards the use of the platform are unclear.

Jamshidi et al. [72] used machine learning (ML) techniques to generate a series of biomarkers (MCP-1 monocyte chemoattractant protein, resistin, and adiponectin) and showed the superiority of the Gradient Boosting Algorithm (GBA) in terms of MSE (Mean Squared Error) for resistin and adiponectin in training and test sets (Table 7). Moztarzadeh et al. [78] reported results similar to Jamshidi et al. [72], showing promising results when RFR (Random Forest Regression) and GBA were used to detect breast cancer. The solutions presented could be useful for shaping the methodology of synthetic data generation for virtual clinical trials; however, without validation in the context of real-world measurements, their usefulness has no practical utility.

Peterson et al. [77] reported TumorScope Predict (TS) as a measure to forecast the tumor response to neoadjuvant therapy (NAT) in patients with early stage breast cancer. The reported overall accuracy of 91.2% of predicting pCR and RD suggests that TS can help to optimize chemotherapy regimens and inform treatment decisions for precision oncology [77].

Christenson et al. [81] implemented orthogonal decomposition (POD) to reduce the computational time required to calibrate a mathematical model for predicting breast cancer response to chemotherapy. The high reported accuracy (Table 7) supports the suitability of ROM (reduced order model) for clinical applications requiring rapid decision making [81].

#### 4.1.2. Lung Cancer

Zhang et al. [57] presented vulnerability detection and its importance in DTHs and VTHs and demonstrated that the Bi-LSTM model with self-attention significantly improves the detection of vulnerable functions in software projects related to lung cancer, outperforming the traditional methods. However, the reported performance was modest (Table 7) and does not support its clinical use.

Qi and Cao [61] simulated a virtual clinical trial on how patients with NSCLC (non-small-cell lung cancer) may benefit from personalized pembrolizumab treatment beyond progression. The authors emphasized the importance of understanding within-patient heterogeneity in treatment responses, particularly for metastatic diseases. These results support the possible usefulness of personalized treatment.

Zhu et al. [65] used an electrical impedance tomography (EIT) lung monitoring scheme and deep learning algorithms (IR-Net) to improve the accuracy of image reconstruction. The proposed IR-Net outperforms the traditional algorithms in terms of accuracy and noise resistance (Table 7) but requires external validation.

Tai et al. [71] constructed a digital twin (DT)-enabled Internet of Medical Things (IoMT) system as a telemedical simulation for patients with lung cancer and pulmonary embolism (PE). The model demonstrates high robustness and accuracy in classifying patients with lung cancer with PE (Table 7) and highlights the need to optimize network transmission demands and improve equipment utilization.

Kolekar et al. [76] introduced a digital-twin-based integrated precision medicine web-service platform called CompMed, designed for personalized medical AI services. The reported performance regarding the 5 year survival prediction for patients with lung cancer showed good performance metrics (Table 7). The key feature of the CompMed platform is the availability of a broad spectrum of medical parameters; however, its inclusion in the daily flux of healthcare needs to be evaluated.

Kolokotroni et al. [83] developed a multidisciplinary hypermodeling scheme (cell kinetics, metabolism, signaling networks, and biomechanics) for the optimization of treatment strategies. The performance of the proposed system, which incorporates clinical, treatment, imaging, and genomic data, is demonstrated in a limited number of real-cases of lung and Wilms tumors.

#### 4.1.3. Gastrointestinal Cancers

Raja et al. [75] investigated the role of adjuvant therapy after neoadjuvant therapy for locally advanced esophageal cancer. While most patients may not benefit, a therapeutic benefit is observed in patients with persistent nodal disease or deeper tumors without nodal involvement. The reported results support the need for individualized treatment plans based on patient and cancer characteristics.

Servin et al. [69] proposed microwave ablation patient-specific surgical planning for liver cancer. The ablation volumes contain different percentages of tumor tissue at frequencies of 915 MHz and 2450 MHz (Table 7), highlighting the importance of tumor properties in ablation forecasting.

Joslyn et al. [82] discussed the development of a quantitative systems pharmacology (QSP) model for TCR-engineered T cell therapy targeting HPV-associated epithelial cancers. The reported results suggest that stem-cell-like memory T cells (T_scm_) are critical for both the expansion and the persistence of TCR-engineered T cells.

Meng et al. [74] introduced a VT metagenome platform to identify functional deviations in the microbiota of patients with colorectal cancer. The performance was good and effective at identifying differential abundance but did not directly establish causality.

Lin et al. [62] used Markov models to simulate the overdiagnosis of FIT screening for CRC in a large population, with an overdiagnosis rate presented in the scientific literature. The researchers emphasized the role of the disease natural history model in distinguishing between non-progressive and progressive cancers, allowing for a more accurate estimation of overdiagnosis.

Mosch et al. [68] presented a two-step method for optimizing neoantigen vaccine composition, utilizing digital twin simulations of a cancer cell population with real data for a limited number of patients with gastrointestinal cancer. The results support a better selection of confirmed neoantigens compared to traditional ranking-based approaches in real patients.

#### 4.1.4. Other Cancers

Kim et al. [73] developed and validated a DT model to predict pathology and biochemical recurrence in patients with prostate cancer. The proposed DT-based model outperformed the traditional methods (Table 7). Despite the significantly high accuracy of the reported model, its clinical applicability is limited, because its performance values are lower than 90%. Chang et al. [79] reported a digital twin (DT) framework to enhance adaptive proton stereotactic body radiation therapy (SBRT) for prostate cancer, addressing uncertainties due to interfractional anatomical variations. The reported results support its clinical use in treatment planning by reducing clinical target volume setup uncertainty, however, clinical validation is needed.

Sharma et al. [80] reported the CervixNet, a classifier model of Pap smear with high accuracy (98.91%). According to our definition, the technology reported by Sharma et al. [80] is not a DTH *per se*, rather it is a DT pathophysiologist. The proposed system has high computational demands and is unable to integrate real-time data, thereby limiting its clinical utility.

Chaudhuri et al. [49] proposed an optimized patient-specific radiotherapy regimen for high-grade gliomas (HGG) under uncertainty. The results have demonstrated that a personalized treatment could extend the median time to tumor progression and survival compared to the standard of care [49]. However, further clinical trials are required to validate the reported results. Pérez-García et al. [70] reported a mathematical model that describes the response of low-grade (WHO grade II) oligodendrogliomas (LGO) to TMZ chemotherapy. The results of this study suggest that the proposed chemotherapy schedules can have equivalent or better long-term efficacy than standard 28 day cycles, but the reported improvements are not necessarily credible (e.g., survival improvement of 68 years).

Susilo et al. [51] investigated digital twin technology to understand clinical dose–response relationships and to identify predictive biomarkers in non-Hodgkin lymphoma (NHL). Individualized virtual patients (iVPs) were treated with increased doses of mosunetuzumab, and the results were promising, with a higher proportion of patients achieving at least a 50% reduction in tumor size by day 42. The proposed solution requires appropriate validation to support clinical implementation.

Bahrami et al. attempted to determine an optimal solution for cancer-related pain management [50,66,67]. The reported results obtained from *in silico* trials support the use of digital-twin-assisted therapy to reduce the average pain intensity and to increase the median time without pain compared to the conventional therapy (Table 7). The effect of individual diversity on cancer-related pain management has been demonstrated using an *in silico* skin model for drug penetration to support personalized treatment [67].

Ahmadian et al. [59] reported a digital twin framework using ReconGAN to simulate the vertebroplasty (VP) procedure, focusing on its impact on the mechanical stability of the vertebrae in cancer patients. The simulations showed that a higher injection flow rate led to a more irregular cement distribution, increasing the risk of intraspinal cement leakage. The simulations showed the impact of the vertebral macroscopic shape and microstructural details on vertebral fracture response [60]. The findings suggest that digital twin frameworks can be a valuable tool for optimizing vertebroplasty procedures and minimizing postoperative complications, as well as for predicting the fracture risk in the presence of spinal metastasis.

### 4.2. Challenges and Limitations of Proposed Technical Solutions

The promising potential of DTHs and VTHs is overshadowed by several challenges that must be addressed. First, creating and maintaining twining solutions requires significant computational, human, and financial resources. Despite the potential benefit of twinning solutions in the management of patients with cancer, the cost of developing and implementing such solutions must be balanced against the benefits. Second, the digitalization of medical- and health-related data is essential, at least for DTHs. A DTH or VTH solution does not exist in the absence of all individual digital, personal, healthcare- and health-related data. In this regard, there are multiple challenges, from the digitalization of healthcare and medical records to obtaining access to such data and an individual’s willingness to share their data. Third, appropriate validation is needed prior to the translation of the twining technologies into standard of care. In the absence of clinical validation, DTHs and VTHs are expensive technical solutions that will be lost in drawers or a waste of money when the outcomes of funded scientific projects will not be implemented in current practice. Fourth, the end-users’ acceptance is a must for its implementation, therefore, the implication of these in all steps of development is crucial.

Synthetic medical data, which are artificially generated healthcare data, are a common tool used in modeling healthcare phenomena, especially in the context of artificial intelligence and twinning [90]. Depending on the availability of real-world data, two different approaches have been used to generate healthcare data. Synthetic data can be generated based on the centrality and dispersion metrics of a limited number of cases with unbalanced data (e.g., limited occurrence of the event of interest) or input requirements imposed by the researchers, in the absence of real data, because of their sensitive nature [91]. Synthetic healthcare data offer certain benefits, but often cannot capture the inherent variability of real-world patient populations. Consequently, the models trained on synthetic data may exhibit lower accuracy when they are applied in clinical settings [92]. The identification and use of diverse real-world sources of healthcare data could provide a reliable solution for DTHs and VTHs. This approach enhances the generalizability of the model and ensures better outcomes across various patient demographics and specific characteristics. As presented in Table 6, some of the DTHs and VTHs analyzed in this manuscript do not use real data or use a limited number of real patients to generate healthcare data. In this context, the main challenge faced by the reported solutions is related to the validity of the proposed technical solutions in clinical scenarios. In this context, Peterson et al. [77] acknowledged the single-site source of data and the use of pretreatment MRI scans as a limitation of their study, which may not be available for all patients or in any healthcare setting. Yankeelov et al. [63] highlighted that the integration of machine-learning- and mechanism-based modeling is a promising approach for future personalized trials. As expected, heterogeneity among individuals and their response to therapy, regardless of the type of cancer, are highlighted as challenging (Peterson et al. [77], Servin et al. [69], Joslyn et al. [82], Qi and Cao [61], etc.).

As DTHs and VTHs are relatively new research fields, real communication must be established between the developers and the end-users (physicians) from the earliest stages of DTH and VTH ideas to support clinical implementation. With no exception, all proposed solutions require validation in clinical settings. The success of DHTs and VHTs is possible only if the end-users and the clinicians know and understand the meaning of *in silico* medicine (e.g., DHT, VHT, personalized medicine, patient-specific modeling, *in silico* clinical trials, and similar expressions) [93], how they work, and the associated clinical-evidence-based usefulness, in order to adopt and accept them as standards of care. The source of healthcare data is expanded as digital endpoints [94] become available at a cost. Sensor-generated data collected outside of the medical setting and capturing patients’ routine living will become a source of data for predicting health conditions. In addition, the inability to fully account for the primary or secondary resistance mechanisms of QSP models (e.g., Susilo et al. [51]) also needs to be technically and scientifically addressed. The world of clinical trials will change with the consideration of AI + humans rather than AI vs. humans scenarios [95,96]. The latest guidelines lead researchers in regard to writing protocols (e.g., SPIRIT-AI [97]), evaluating AI-prediction models (TRIPOD + AI [98,99]), and reporting their results in the scientific literature (CONSORT-AI [100]).

Real-world evidence [101] and AI-based deep precision medicine [87] are expected to find their place in medicine and healthcare in the near future. The use of DTHs and VTs is intended to be crucial for practical implementation. A ‘digital twin’ (DT) is a digital replica of a patient that integrates real-time data, enabling continuous monitoring and intervention. In contrast, a ‘virtual twin’ (VT) is an active simulation designed to test various therapeutic scenarios without requiring a real-time update. Both technologies offer significant benefits in oncology, but their applications differ based on the need for real-time intervention versus testing hypothetical scenarios. In this new environment, twinning will have its place [32,102,103,104], inclusive towards precision oncology [42,105,106].

### 4.3. Ethical Challenges

Using DTHs and VTHs is supposed to be beneficial for patients, assisting in personalized treatments plans, leveraging personal history files, analyzing real-time data, and providing personalized healthcare solutions. The main ethical and legal concerns raised by scientists and ethicists in the use of DTHs and VTHs refer to personal data privacy, technical effectiveness and accuracy, decision responsibility, inequalities in healthcare access, and the idea of a digital or virtual twin as a clone of a person.

Ethical concerns related to personal life and privacy are especially focused on sensitive and personal data shared with third parties and the risk of using data against patients’ interests [107]. DTHs and VTHs are fed a large amount of personal data, including biological, genetic, physical, and personal lifestyle data. The ethical question is as follows: What happens with all data? They can be destroyed, anonymized, secured, or kept for further use, by private or public organizations. These organizations might have their own interests or benefits and so not protect the individuals’ benefits [107], which is the key concept and the main concern of personal life and privacy protection by using DTHs and VTHs in healthcare practices. In addition, there is an increasing risk of ransomware and blackmailing if the digital twins are not sufficiently protected from hackers and patients’ health data become public information. To protect patients’ personal data and ensure an ethical design and use of DTHs and VTHs, ethicists must be involved from the beginning of the project. Questions and ethical dilemmas must be continuously addressed from the earliest steps, namely before validation and implementation in healthcare practices. The earliest address of possible ethical issues will lead to patients, community, and regulatory bodies’ confidence and trust in the new technologies and will ensure the transparency of the design process.

The declared aims of DTHs and VTHs are to do good, to help healthcare professionals and patients make informed decisions, and to avoid confusion and misuse of limited resources in healthcare. These aims are supported by the effectiveness and accuracy of solutions. When human judgement is replaced with artificial intelligence, it is no longer about the judging process but about the capacity to compute. Thus, the truth is replaced by formal correctness. Consequently, what was formally correct may not be true. ‘Even a lie can be correct’ [108]. As VTHs can run multiple simulations to assess multiple processes, and not one particular simulation related to one process, it is impossible to verify the outcome in terms of efficiency and accuracy. This is the main issue and concern raised by using VTHs in healthcare practices, as follows: providing treatment plans for patients based on DTHs or VTHs assessment, without explaining why this treatment is the best option for the patient.

The use of DTHs and VTHs in screening, diagnosis, prognosis, treatment, prediction, and monitoring of oncologic patients raises the following questions: Who would be held responsible if something went wrong? Who is the person who created the patient prejudice or harm: healthcare practitioners (doctors, nurses), healthcare providers, producers, owners, insurers, or patients? Could a manufacturer of a DTH or VTH be held liable for medical malpractice or misuse of personal data? The physician will face a huge amount of data and has no possibility of interpreting it, handling it [109], or even understanding it. As a direct consequence, the patient has no possibility of proving negligence or errors and is at risk of seeking a legal remedy [109]. In addition, it would be impossible to proceed with medical expertise to identify the cause of patient prejudice.

The twins are modeled based on the input data provided by the developers. In some cases, these data refer to white, healthy, middle-aged males [107]. Solutions and results for pre disease, disease, and post disease management are generated based on the input data and will reflect the targeted population. Consequently, patients other than those in the targeted group are excluded from personalized medicine access, inducing inequalities in healthcare access.

Digital and virtual twins are perceived as loopholes through which people can be cloned [109]. Consequently, digital clones may take over the control of personal data, including the healthcare information of individuals. They may then predict and manipulate decisions, in real time, by using the individuals’ data against their interests and benefits [110]. Human cloning is prohibited by international legal framework, as follows: the United Nations Declaration on Human Cloning (2005) [111] and the European Patent Convention (1973) [111], in Article 53, forbid the granting of a patent to inventions that breach public order or morality, as well as The Court of Justice of the European Union, in *Oliver Brüstle v Greenpeace eV*. Case [112]. Legal orientations argue in favor of the protection of fundamental rights [113], such as human dignity, integrity, free choices, and autonomy. The analogy between DTHs or VTHs and clones is debatable and, from this point of view, some voices state that it should also be banned.

Other possible ethical concerns are related to the newly emerging type of doctor–patient relationship. Digital and virtual twins become intermediate between healthcare professionals and patients, with the consequence of losing autonomy and control in decision making, both for patients and healthcare professionals. Twins that are designed to help and assist healthcare practice could bring more confusion and fear for patients and create many more responsibilities for healthcare professionals to change the medical protocols by introducing DTH and/or VTH in diagnosing the disease and in the treatment plan. By informing patients about the benefits and risks of using DTHs and/or VTHs, the patients’ refusal to use these technologies could lead to the absence of alternatives to medical care. If patients accept an investigation using twins, there are concerns about the access for all patients or for only some patients, such as citizens or patients who can financially afford them, which raises the reconsideration of patients’ rights in terms of equality to access this innovation [114].

Protecting patient privacy is crucial for preventing data misuse and maintaining trust in DTH and VTH technologies. Concerns about “digital cloning” highlight the need for clear distinctions between data replication and the creation of autonomous digital identities. Additionally, there is a risk of exacerbating the inequalities in healthcare access if these advanced tools are available to only certain demographics. Developing policies to ensure equal access and the ethical use of these technologies is critical as they continue to evolve.

### 4.4. Study Strengths and Limitations

To the best of our knowledge, this is the first scoping review that reports the latest advancements in DTHs and VTHs in oncology, highlighting their technical components and medical applicability. A comprehensive search that went beyond medical bibliographic databases and included relevant research projects was performed. The search was not limited to the retrieved items, and the reference lists of the identified reviews, editorials, perspectives, and preprints were also sources of bibliographic documentation. Despite our efforts, we doubt that all of the relevant manuscripts and unpublished materials have been identified. Additionally, the applicability in oncology is a relatively new growing field, so it is expected to have unstandardized terms and keywords. Therefore, the possibility of missing relevant contributions must be highlighted. The detailed description of the reported technical solutions and the assessment of fairness and credibility are the strengths of this scoping review. Please note that this manuscript reflects the expertise of all of the researchers involved. As per the nature of the reported technical solutions, the type of study, and the absence of clinical validation, recommendations for standards of care cannot be provided.

### 4.5. Future Research Directions

The reported DTH and VTH technologies show promising capabilities in oncology; however, the evaluated solutions lack appropriate clinical validation. This lack of validation restricts their adoption in standard clinical settings, underscoring the need for clinical trials to establish their efficacy, safety, and reliability in diverse patient populations. Although DTH and VTH applications have demonstrated success on a small scale, there are significant technical and resource-related barriers to their scaling. The limitations in computational power, data integration from multiple sources, and interoperability on one hand, and understanding their utility, acceptance, and ethical use on the other hand, are key aspects that must be addressed in order to broaden their applicability in real-world, large-scale settings.

The potential of DTHs and VTHs to revolutionize cancer treatment is huge, so there are multiple research directions. First, DTHs can aid early detection by analyzing patient data and identifying the potential signs of cancer before they become symptomatic. Access to reliable personal health data and medical history in a digital format is a requirement for personalized prediction with DTHs. Second, by creating a digital replica of a patient with cancer, physicians can simulate different treatment options, predict outcomes, and tailor therapies to the individual patient’s unique characteristics with VTH solutions. Through simulations, physicians can test various treatment combinations, dosages, and sequencing in a safe virtual environment to determine the most effective approach for a specific patient. However, what the VTH will retrieve as the optimal treatment schema must be validated; therefore, research to translate the virtual outcome into a real-world settlement must be validated. By understanding a patient’s individual response to treatment, healthcare providers can minimize adverse effects, improve the overall quality of life, and maximize the use of financial resources and medications. Moving outside of the individual patient, VTHs can revolutionize drug development using *in silico* approaches for the identification and assessment of new active compounds, offering a safe and less expensive environment for drug production. Such approaches can be useful to simulate drug trials, reducing the time and costs associated with traditional clinical trials with immense potential for training and optimizing treatment strategies. Third, existing DTH and VTH solutions must be adopted by medical staff. The principles of technology adoption are vast and include the following [115]:

- Partnership with the end-users from the early steps of technology design and development;
- Interoperability between and within the existing digital solutions, including EHRs (electronic health records), EMRs (electronic medical records), imaging devices (MRI, CT, mammography, and echography), medical analyses, and e-prescription;
- Appropriate connectivity, including tele-expertise and tele-consultation;
- Trustworthiness by demonstrating that the solutions are reliable and work appropriately in clinical settings;
- Beneficiary by showing the users the benefits of using such technologies;
- User-friendliness, including the ease of use and clinician confidence in using the technology;
- Evaluation to assess use and user satisfaction, as well as the benefits of quality of life for the individual patient, access to the individual and healthcare staff, and the productivity for healthcare organizations.

An emphasis on precision, data integration, and clinical utility is essential for the successful development of DTH and VTH technologies in oncology. These solutions are expected to focus on patient-specific modeling and precision medicine, a strategy that requires high-quality patient data with dynamic, real-time updates to support dynamic adjustments and more accurate outcomes. Interoperability and aggregation of data from different sources (e.g., EHRs, EMRs, imaging, labs, patient-generated data, etc.) require the appropriate investment for appropriate integration. The analysis of a large amount of data or simulations requires a scalable computational infrastructure or access to cloud computing solutions. Computation infrastructure or access to such infrastructure is expensive and unaffordable for all healthcare providers, opening the pathway towards inequalities in healthcare for people around the world. DTH and VTH solutions require robust clinical validation and evidence generation. Appropriate validation in clinical settings and critical appraisal of the results must be conducted to ensure that such solutions can reliably predict the outcomes. Guidelines on using sensitive oncology data ethically and transparently—particularly genetic and tumor data, which can be sensitive and complex—are needed to support the development and implementation of DTH and VTH technologies in oncology. Furthermore, DTH and VTH technologies must meet the required standards for patient safety and data security (e.g., FDA for US clinical devices, and GDPR in Europe), as well as medical device regulations for inclusion in daily clinical activity.

To enhance the usability and adoption among oncology clinicians, the end-users must be engaged in the design process to ensure that the twins’ interface meets the specific needs of cancer care and fits within existing oncology workflows. Education and training must be provided for oncologists and oncology teams to build trust in the twining utility, helping them to appropriately interpret the model and simulation outputs, along with their limitations.

Twinning solutions require a large amount of high-quality data; therefore, partnerships with cancer centers, research institutions, and technology companies to facilitate data sharing, resource pooling, and large-scale validation are desired. Furthermore, sharing access to DTH and VTH solutions could ensure a timely translation of existing solutions to more broadly accessible solutions across the oncology field. Actions toward the education and engagement of patients regarding DTH and VTH benefits will pave the way for acceptance. Enhancing patient trust and engagement can be achieved if the patients understand how digital twins can support their treatment journey. Patient involvement in model development, particularly for monitoring solutions, will ensure that DTH and VTH applications are aligned with their needs and concerns.

Interdisciplinary collaboration is essential to bridge the gaps between technology developers, end-users, and healthcare providers, ensuring that the solutions meet the practical clinical and personal needs.

## 5. Conclusions

The use of twinning solutions in oncology is a relatively new field with a paucity of robust evidence. The reported evidence lacks clinical validation, as most does not respect the dissemination guidelines, as per medical journals, and does not discuss the ethical implications. Overall, the reported technical solutions lack transparency, visibility, and replicability. However, the solutions are promising for patient-centered care in oncology.

Although DTH and VTH technologies have shown potential in oncology, most of the existing solutions have not undergone thorough clinical validation. This gap limits their adoption in daily practice, highlighting the need for rigorous trials to verify their efficacy, safety, and reliability in diverse patient populations. Despite demonstrating success on a smaller scale, DTH and VTH applications face significant scalability challenges. The key issues include limitations in computational power, data integration from multiple sources, and interoperability, as well as understanding their utility, acceptance, and ethical use. Addressing these factors is essential for expanding their applicability in daily healthcare practice.

## Figures and Tables

**Table 1:** Three-layer digital twin architecture and characteristics by example.

Layers	Key Components
Hardware: physical components like IoT sensors, actuators, edge servers, and routers that collect real-time data from the physical asset or system.	Data platform: storing and processing large amounts of data using cloud services and performing analysis (using AI and ML).
Middleware (data processing): data governance, integration, visualization, modeling, connectivity, and control.	Visualization: translating the data into formats suitable for human perception, creating a connected environment between the virtual and physical worlds.
Software: analytics engines, machine learning models, and data dashboards to analyze the data and generate insights.	Workflow and APIs: synchronizing the digital twin with its physical counterpart by pulling and sharing data from different sources.
	Governance and operations: ensuring proper data structure, availability, and value delivery.

AI = artificial intelligence; ML = machine learning; API = application Programing Interface.

**Table 2:** Digital twin vs. virtual twin characteristics.

	Digital Twin (DT)	Virtual Twin (VT)
Digital replica	Mirror of real-life versions of the patient.	Virtual high-detailed model of a patient designed for simulation and testing in a virtual environment.
Application	Show what happens now and what may happen in the future.	Simulate potential scenarios to show the targeted outcomes based on changes in input data.
Key technologies	IoT devices, real-time data analytics, artificial intelligence (AI), and machine learning (ML).	Virtual reality technologies, simulation models, artificial intelligence (AI), and machine learning (ML).
For…	Patient care monitorization → personalized diagnostic, treatment, monitorization, etc.	Decision of the best personalized healthcare intervention → personalized healthcare. Training medical staff to deliver healthcare for a specific patient → personalized healthcare and personalized training for medical staff.
Interactivity	Real-time and dynamic interaction with the real world (e.g., IoT devices), the user monitor, and receives data.	Active engagement: the users might adjust parameters, change conditions, or even interact with the virtual environment directly.
Data flow	Bidirectional: physical patient ↔ virtual patient, real-time updates, and interventions.	Unidirectional: physical world → virtual model.
Value	Monitoring and real-time decision prediction.	Establish the designs without the costs and constraints of real-world experimentation.
Allow	To understand the current status and to forecast potential issues.	To evaluate hypothetical scenarios to simulate potential outcomes based on specific inputs.
Challenges	Technical: data (integration, harmonization, standardization, storage, interoperability, security, etc.), computing resources (high-performance and advanced infrastructure), skilled researchers, etc. Modeling: data (collection, reliability and validity, representativeness, etc.), optimization, risk of learning from biased data, updating with real-world data, etc. Ethical: data privacy and security and national and international regulatory laws.	Technical: advanced hardware for implementation of highly detailed virtual platforms, specific expertise, technical skills, etc. Virtual representation: advanced software, high-fidelity data, computing resources, highly qualified technical skills, and rules for validation of optimal solutions. Ethical: national and international regulatory laws.
Disadvantages	Constraints of real-time data or physical prototypes. High implementation and maintenance costs.	Evaluation is needed for the identified best performing approach. The implementations and maintenance costs are high.
Example	DT for real-time monitoring of radiotherapy response (imaging and clinical measurements) and dose adjustment based on patient response.	VT model of a new painkiller-drug-delivery implantable device for patients with cancer → testing the device under various scenarios (e.g., unique characteristics of the patients, doses, administration procedures, etc.) to decide the appropriate surgical solution for the enhancement of safety and efficacy.

**Table 3:** Search sources and used strings.

Database	Search String	Filters
PubMed	(cancer OR oncology) ((virtual twins) OR (digital twins))	Species = Humans and Article language = English
WoS	(cancer OR oncology) ((virtual twins) OR (digital twins)) (topic)	Document type = Article or Meeting or Dissertation Thesis AND Language = English
Scopus	(cancer OR oncology) AND ((virtual AND twins) OR (digital AND twins))	Document type = Article or Conference paper AND Language = English

WoS—Web of Science; Scopus search was conducted within the article title, abstract, and keywords.

**Table 4:** Websites of projects dealing with digital or virtual twins addressing oncology.

Website	Who?	Search String
https://dth.openaire.eu/search/advanced/research-outcomes (accessed on 19 August 2024)	European Virtual Human Twin project (EDITH) (https://www.edith-csa.eu/ (accessed on 19 August 2024))	digital twins cancer * virtual twins cancer *
https://www.certainty-virtualtwin.eu/ (accessed on 19 August 2024)	CERTAINTY project	browse the Results Section
https://frederick.cancer.gov/news/digital-twins-cancer-care-exploring-cross-disciplinary-innovative-approach (accessed on 19 August 2024)	Frederick National Laboratory for Cancer Research	browse the article list of the principal investigators of scientific funded proposals

* Publications.

**Table 5:** Summary of the evaluated evidence.

Reference	Year	Country	Type
Zhang et al. [57]	2020	China	Conference paper
Meraghni et al. [58]	2021	France, Algeria	Conference paper
Ahmadian et al. [59]	2022	USA	Journal article
Ahmadian et al. [60]	2022	USA	Journal article
Qi and Cao [61]	2023	USA	Journal article
Lin et al. [62]	2023	Taiwan	Journal article
Bahrami et al. [50]	2023	Switzerland	Journal article
Yankeelov et al. [63]	2023	USA, Italy	Journal article
Gamage et al. [64]	2023	New Zealand	Conference paper
Zhu et al. [65]	2023	China, UK	Journal article
Bahrami et al. [66]	2024	Switzerland, Belgium	Journal article
Bahrami et al. [67]	2024	Switzerland	Journal article
Mosch et al. [68]	2024	Germany	Journal article
Servin et al. [69]	2024	USA	Journal article
Pérez-García et al. [70]	2024	Switzerland, Spain, Mexico	Journal article
Tai et al. [71]	2022	China	Journal article
Jamshidi et al. [72]	2022	Czech Republic, Iran	Conference paper
Kim et al. [73]	2022	South Korea	Journal article
Meng et al. [74]	2023	China, USA	Journal article
Raja et al. [75]	2023	USA	Journal article
Kolekar et al. [76]	2023	South Korea	Conference paper
Susilo et al. [51]	2023	USA	Journal article
Peterson et al. [77]	2023	USA	Journal article
Chaudhuri et al. [49]	2023	USA, Italy	Journal article
Moztarzadeh et al. [78]	2023	Czech Republic, Iran	Journal article
Chang et al. [79]	2023	USA	Preprint article
Sharma et al. [80]	2024	India, UK	Journal article
Christenson et al. [81]	2024	USA	Journal article
Joslyn et al. [82]	2024	USA	Journal article
Kolokotroni et al. [83]	2024	Greece, Switzerland, USA, Ireland, UK, Germany	Journal article

USA—United States of America; UK—United Kingdom.

**Table 6:** General characteristics of the evidence.

Reference	Study	Cancer	No. Patients	Twin of…	Intervention	Outcome
Zhang et al. [57]	Simulation	Lung-PE	na	OBO	Monitoring	Accuracy
Meraghni et al. [58]	Simulation	Breast	na	OBO	Diagnostic	Accuracy
Ahmadian et al. [59]	Feasibility	Metastasis +	na	OBO	Therapy	Efficiency
Ahmadian et al. [60]	Feasibility	Lung metastasis +	1	OBO	Therapy	Accuracy
Qi and Cao [61]	Simulation	Lung	524	OBO	Therapy	Patient outcome
Lin et al. [62]	Simulation	Colorectal	na	OBO	Diagnostic	Accuracy
Bahrami et al. [50]	Simulation	Pain	20	WB	Therapy	Patient outcome
Yankeelov et al. [63]	Simulation	Breast	1	OBO	Therapy	Patient outcome
Gamage et al. [64]	Feasibility	Breast	922	OBO	Diagnostic and Therapy	Patient outcome
Zhu et al. [65]	Simulation	Lung	17	OBO	Monitoring	Accuracy
Bahrami et al. [66]	Simulation	Pain	8	WB	Therapy	Patient outcome
Bahrami et al. [67]	Simulation	Pain	na	WB	Therapy	Patient outcome
Mosch et al. [68]	Simulation	Gastrointestinal	7	FBC	Therapy	Efficacity
Servin et al. [69]	Simulation	Liver	4	OBO	Therapy	Efficacity
Pérez-García et al. [70]	Simulation	Brain *	11	OBO	Therapy	Efficacity
Tai et al. [71]	Feasibility	Lung	1462 ^a^	OBO	Diagnostic	Accuracy
Jamshidi et al. [72]	Feasibility	Breast	116 ^b^	OBO	Diagnostic	Accuracy
Kim et al. [73]	Simulation	Prostate	404	OBO	Diagnostic	Accuracy
Meng et al. [74]	Simulation	Colorectal	771 ^c^	OBO	Diagnostic	Accuracy
Raja et al. [75]	Simulation	Esophageal	9079 ^d^	OBO	Prognostic	Patient outcome
Kolekar et al. [76]	Feasibility	Lung	4591 ^e^	WB	Therapy	Accuracy
Susilo et al. [51]	*in silico* trial	Blood **	140	OBO	Therapy	Efficacy
Peterson et al. [77]	Simulation	Breast	80	OBO	Therapy	Accuracy
Chaudhuri et al. [49]	Simulation	Brain *	na	OBO	Prognostic	Patient outcome
Moztarzadeh et al. [78]	Simulation	Breast	116 ^f^	OBO	Diagnostic	Accuracy
Chang et al. [79]	Simulation	Prostate	10	OBO	Therapy	Accuracy
Sharma et al. [80]	Simulation	Uterus	na	OBO	Diagnostic	Accuracy
Christenson et al. [81]	Simulation	Breast	50	OBO	Therapy	Accuracy
Joslyn et al. [82]	Simulation	Pancreas	10	WB	Therapy	Efficacy
Kolokotroni et al. [83]	Simulation	Kidney *** and Lung	3 ^g^	WB	Therapy	Efficacy

NSCL—non-small-cell lung cancer; PE—pulmonary embolism; + vertebral metastasis; * (oligodendro)glioma; ** non-Hodgkin’s lymphoma; *** Wilms tumor; na = not available; ^a^ 90 patients with PE and 1372 patients without PE; ^b^ 64 patients and 52 healthy controls; ^c^ 387 patients and 384 healthy controls; ^d^ 7731 patients with adenocarcinoma or squamous cell carcinoma who received neoadjuvant therapy and 1348 patients who received additional adjuvant therapy; ^e^ NSCLC (non-small-cell lung cancer) patients, which comprised 3470 cases, and SCLC (small-cell lung cancer) patients, which comprised 1121 cases; ^f^ 64 patients and 52 healthy controls; ^g^ 1 63-year-old women with lung cancer and 2 children with Wilms tumors; OBO—one body organ; WB—whole body; FBC—finer body component levels (cellular and subcellular).

**Table 7:** Characteristics of what has been carried out and the main reported results.

Reference	Twin for…	What Was Investigated	Reported Results
Zhang et al. [57]	Tumor behavior	“Cyber resilience” towards operational capacity and reliability under cyberattacks.	DeepVR vs. LSTM in open-source dataset precision: 0.78 vs. 0.69, recall: 0.77 vs. 0.63, and F1 score: 0.78 vs. 0.62.
Meraghni et al. [58]	BC diagnostics	Bio-heat model for different levels of fat in the breast, external temperature, and blood perfusion rate.	Line and column graphs showing skin temperature.
Ahmadian et al. [59]	Simulating the vertebroplasty	Mechanical integrity of the vertebral body in a cancer patient with a lytic metastatic tumor.	A total of 72% strength recovery expected for 6.0 mL of cement injection.
Ahmadian et al. [60]	Whole vertebra model	Deep convolutional generative adversarial network to generate trabecular microstructure.	Impact of the vertebra macroscopic shape and microstructural details on the VF response.
Qi and Cao [61]	VCT for NSCLC therapy	Lesion-level response dynamic under 18 weeks of chemotherapy.	Real vs. simulated ORR: chemotherapy: 31.4% vs. 29.7%; and pembrolizumab: 44.3% vs. 41.6%.
Lin et al. [62]	Overdiagnosis of FIT	Markov algorithms for FIT in screening of CRC.	Overdiagnosis: invasive cancer 4.16% (with adenoma) vs. 15.83% (without adenoma).
Bahrami et al. [50]	Personalized fentanyl transdermal therapy—DTAT	Markov chain Monte Carlo—MCMC with seven parameters considering sex, weight, and height.	Fentanyl concentration in plasma increased by 11.5% and average minute ventilation decreased by 15% with DTAT. Pain intensity < 3VAS: 98.8% vs. 57.1% DTAT vs. ConT.
Yankeelov et al. [63]	Therapy BC	‘‘Biology-based’’ model biological mechanisms underlying the growth and treatment response of cancer based on imaging.	SOC vs. ALT protocols: predicted median drug concentration in the healthy breast tissue ^#^: 79 and 81 days; predicted TV: 5.69 and 2.68 cm^3^.
Gamage et al. [64]	Computational physiology models	Prototype of the 12 LABOURS Digital Twin Platform.	Portal demonstration for clinical breast MRIs, 922 patients.
Zhu et al. [65]	EIT-based lung	Framework for EIT image reconstruction.	↓RIE, ↑SSIM, ↑CC compared to other methods on data with and without noise.
Bahrami et al. [66]	Patient physiology	Personalized switch from oral/IV morphine to transdermal fentanyl.	Morphine therapy vs. fentanyl patch—max conc in plasma: 26 to 61 nM vs. 1.1 to 2.5 ng/mL; min pain intensity: 3.2 to 4.4 VAS vs. 0.5 to 3.3; min minute ventilation: 10.4 to 15.6 L/min vs. 2.7 to 10.1 L/min evaluated physiological features correlated (>0.9) with weight.
Bahrami et al. [67]	*in silico* skin model for drug penetration	Effects of skin characteristics at application sites in fentanyl transdermal therapy.	c_max_ (ng/mL): 1.333 (flanck), 1.176 (back), 1.170 (upper arm), 1.156 (chest) t_max_ (h): 19.8 (flanck), 27.1 (back), 28.2 (upper arm), and 30.1 (chest).
Mosch et al. [68]	Personalized neoantigen vaccine	Vaccine composition optimization on simulating individual cancer cell.	Response probability defined as the likelihood of a simulated cancer cell to be eliminated by a CD8+ T cell, for a given vaccine composition—graphical distributions.
Servin et al. [69]	Patient-specific surgical planning	Image-guided microwave ablation therapy	% tumor tissue from ablated volume increased with MHz and Fat Content Index Tumor (naïve DT vs. tumor-informed DT: 50–68% vs. 45–70% for 915 MHz and 70–90% vs. 80–95% for 2450 MHz).
Pérez-García et al. [70]	*In silico* twins for chemotherapy	Standardizing treatment (proposed) for virtual patients: 5 monthly induction cycles and 12 cycles every three months for maintenance.	Survival improvement: median 5.69 years (from 0.67 to 68.45 years) and survival probability 3.8 years for standardized method vs. random cycles—HR = 0.679 (*p* < 0.001).
Tai et al. [71]	IoMT-based MR simulator	Customized LC with PE Diagnostic Intelligent IoMT through MR.	AUC = 0.93; 12 misclassification six false positive and six false negative classifications.
Jamshidi et al. [72]	Biomarker generation	ML (linear regression and Decision Tree Regression) and Random Forest Regression.	Graphical representations of real and twin values; MSE resistin: 1.768 train and 1.843 test for GBA vs. 4.598 train and 10.640 test for LRM; adiponectin: 1.1518 train and 1.09 test for GBA vs. 2.439 train and 5.56 test for LRM.
Kim et al. [73]	DT-based predictive model	ML for biopsy markers: ECE, SVI, PNI, LVI, SM, Pathology T, SUM, and BCR.	Random forest best performing Acc: 85.4% SVI, 84.7% BCR, 83.2% LVI, 82.2% ECE, 81.7% SUM, and 80.2% Pathology T
Meng et al. [74]	Genetic VT	VT metagenome platform—microbiota feature in diagnosis CRC.	DA-CRC vs. controls: 37.0 ± 20.6 vs. 8.1 ± 5.6, *p* < 0.05. AUC for 30 top species identified by VT: Intra-Cohort CV: 0.89 ± 0.09 (real data); Cross-Cohort Validation: 0.78 ± 0.06; LODO Validation: 0.81 ± 0.06 (0.81 ± 0.08 for all species).
Raja et al. [75]	Virtual-twin survival predictions (VT-SP)	VT-SP: survival benefit in case of adjuvant therapy after neoadjuvant therapy in patients with locally advanced esophageal cancer.	Survival benefit when the patient received adjuvant therapy: 3.2 ± 10 month (adenocarcinoma) and 1.8 ± 11 months (SCC); mean gain in lifetime with adjuvant therapy for patients with significant residual disease burden after neoadjuvant therapy: 22 ± 6.0 months (adenocarcinoma) and 23 ± 8.1 months (SCC).
Kolekar et al. [76]	Five-year survival prediction for patients with lung cancer	Web-services platform towards DTH.	ResNet-18 model (binary outcome) trained with clinical and radiomic features: C-index score = 0.97; MAPTransNet with LCSA: C-index = 0.82, MAE = 260 days; prediction of in-hospital clinical deterioration: F1-score = 0.652, Se = 0.77, AUC = 0.837.
Susilo et al. [51]	VPOP with iVPs for exposure response assessment as per different clinical indications	QSP digital twin for dose/exposure- response and potential pretreatment biomarkers with predictive abilities.	Tumor size ↓ when ↑ doses of mosunetuzumab are used (graphs). Proliferation rates and T-cell infiltration identified as potential markers.
Peterson et al. [77]	Forecast therapy response based on DCE-MRI and patient’s tumor biology	Three-dimensional virtual *in silico* tumor segmentation model based on DCE-MRI and incorporating patient’s tumor demographics and biology.	TS overall Acc = 91.2 [82.8 to 96.4%]. Prediction Acc varied by receptor subtype: 93.8% [55.5 to 99.8%] for TNBC and 75% [68 to 93.2%] for HR+/HER2− EFS 5-year ROR TS-simulated vs. clinical pCR: HR = −1.99 [−3.96 to −0.02], *p* = 0.043 vs. −1.76 [−3.75 to 0.23], *p* = 0.054.
Chaudhuri et al. [49]	*in silico* optimal RT plans	Optimized patient-specific RT in patients with HGG.	RT dose is higher than SOC in optimized treatment for real patients (graphs); optimal RT with doses higher than SOC (60 Gy) show superior survival outcomes (graph, virtual cohort).
Moztarzadeh et al. [78]	Disease extent and progression with metaverse	Biomarkers as input data ML algorithms: LR, DTR, RFR, and GBA.	The closest method to the measured biomarkers is given visually by GBA.
Chang et al. [79]	Planning therapy	Optimal treatment plans for prostate cancer.	Dose volume: ↑ DT-plans vs. SOC; ProKnow scores with range from 1.4% to 10.5% (10 patients).
Sharma et al. [80]	Computer-assisted diagnostic cervical cancer	CervixNet classifier model of Pap smear pictures using ML algorithms: ANN, SVM, RF classifier, k-NN, NN, and NB classifier.	Acc: 98.9% (SVM), 91.8% (RF), 97.8% (k-NN), 95.9% (NN), and 97.5% (NB).
Christenson et al. [81]	Therapy response of patients with triple negative breast cancer	MRI predictions of tumor growth and response to chemotherapy— response to the 3rd and 4th cycles of chemotherapy.	Similar performance of ROM and FOM for the most complex model. Prediction Acc: 0.99 ± 0.0055 for ΔTTV and 0.98 ± 0.0060 for ΔTTC.
Joslyn et al. [82]	Virtual clinical trial	QSP model of T cell cellular kinetics of T_endo_, T_scm_, T_cm_, T_em_, and T_eff_.	*In silico* individual virtual cellular kinetics (cells/mL) of 10 patients treated with TCR-engineered T cells (10^9^, 10^10^, and 10^11^ cells) vs. experimental data (graphs). Graphs: 14 parameter space ridgeline plots, biological variability, % TCT in blood over 365 days, and PRCC for persistent and non-persistent group graphs. Predictive simulation on two patients with pancreatic cancer treated with TCR-engineered T cells targeting KRAS G12D.
Kolokotroni et al. [83]	DT-based clinical decision support system	Two Oncosimulators: Lung (response to external beam radiotherapy) and Wilms tumor (WT) (tumor response to preoperative combined actinomycin and vincristine).	A lower dose (10 Gy) led to a TCP of 0 and a median FTV of 0.91 mm^3^. An early treatment would have similar results with actual treatment (15 Gy) in terms of TCP (median 7 × 10^−12^ vs. 4 × 10^−12^) and FTV (around 0 vs. 0.91 mm^3^). Graphical representation of tumor volume dynamics for two cases with WT and different interventional scenarios. Graphs of tumor volumes (simulated vs. observed) for each real case.

BC—breast cancer; LSTM—long short-term memory; VT—vertebral fracture; ORR—objective response rate; VCT—virtual clinical trial; NSCLC—non-small-cell lung cancer; FIT—fecal immunological test; CRC—colorectal cancer; DTAT—digital-twin-assisted therapy; ConT—conventional therapy; SOC—standard of care; ALT—alternative protocol; TV—tumor volume; EIT—electrical impedance tomograph; RIE—relative image error; SSIM—structural similarity index measure; CC—correlation coefficient; min—minimum; max—maximum; c_max_—maximum concentration of fentanyl in plasma; t_max_—time to reach this maximum concentration; IoMT—Internet of Medical Things; MR—magnetic resonance imaging; ML—machine learning; ECE—extracapsular extension; SVI—seminal vesicle; PNI—perineural invasion; LVI—lymph node metastasis; SM—surgical margin, SUM—Pathology Gleason score, BCR—biochemical recurrence; DA—differential abundance; AUC—area under the receiver operating characteristic curve; CV—Cross-Validation; c—Leave-One-Dataset-Out; SCC—squamous cell carcinoma; LCSA—Survival Analysis Based on Lung Tumor Segmentation; MAE—Mean Absolute Error; Se—sensitivity; VPOP—virtual population; QSP—quantitative systems pharmacology; iVPs—individualized virtual patients; DCE-MRI—dynamic contrast-enhanced magnetic resonance imaging; TS—tumor volumetric response; SOC—standard of care; HGG—high-grade glioma; RT—radiotherapy; LR—linear regression; DTR—Decision Tree Regression; RFR—Random Forest Regression (RFR); GBA—Gradient Boosting Algorithm; ANN—artificial neural network; SVM—support vector machine; RF—random forest classifier; k-NN—supervised k-nearest neighbor; NN—neural networks; NB—naïve Bayes classifier; ROM—reduced order model; FOM—full order model; ΔTTV—change in total tumor volume; ΔTTC—change in total tumor and cellularity; T_endo_—endogenous CD3 + T cells, CD8 + TCR-engineered T cells; T_scm_—stem-cell-like memory T cells, T_cm_—central memory T cells, T_em_—effector memory T cells, T_eff_—effector T cells; TCP—tumor control probability; FTV—final tumoral volume. ^#^ Duration of drug concentrations lower than the empirical toxic concentration. ↑ = increase, ↓ = decrease.

**Table 8:** Technical characteristics of the proposed solution: hardware, middleware, and software layers.

Reference	Middleware	Software
Zhang et al. [57]	Communication layer (IoT sensors) AI models Healthcare system	Unity for VR application; Cybersecurity measures for vulnerability detection.
Meraghni et al. [58]	Data flow	Data processing algorithms (cleaning and transforming data); algorithm for tumor detection; real-time data analysis and decision making
Ahmadian et al. [59]		COMSOL *; DCGAN **
Ahmadian et al. [60]		COMSOL; 3D DCGAN; Python’s scikit-image library for image processing.
Qi and Cao [61]		MATLAB for conducting treatment simulations; Monolix for performing nonlinear mixed-effects population modeling; and WebPlotDigitizer for extracting data from published studies.
Lin et al. [62]		SAS for statistical analysis and the implementation of Markov models
Bahrami et al. [50]		COMSOL ^#^; RStudio
Yankeelov et al. [63]		Biology-based mathematical models.
Gamage et al. [64]	Physiome Workflow Manager and APIs	Gen3 (metadata management); iRODS (data storage); Python-based tools like the Physiome Workflow Manager and Sparc-Me (data processing and management); Three.js-based Scaffoldvuer and Plotvuer libraries (data visualization).
Zhu et al. [65]		U-Net-based image reconstruction neural network (IR-Net) trained on datasets generated from the DT models; the training and implementation are carried out in a PyTorch-GPU environment.
Bahrami et al. [66]		COMSOL Multiphysics for solving the diffusion process of fentanyl through the skin and pharmacokinetics/pharmacodynamics modeling; and RStudio was used for generating the virtual population and analyzing data.
Bahrami et al. [67]		COMSOL Multiphysics for simulating drug uptake, blood flow, and heat transfer; MUMPS solver within COMSOL was used for simulations.
Mosch et al. [68]		Optimization algorithms.
Servin et al. [69]	Simulation integration layer	Finite element method.
Pérez-García et al. [70]		Mathematical models.
Tai et al. [71]	Cloud communication layer	GAN-based predictive models.
Jamshidi et al. [72]	Metaverse middleware	Metaverse AI models.
Kim et al. [73]	Predictive model processing	Machine learning tools.
Meng et al. [74]		Causal inference algorithms and
Raja et al. [75]		random forest model.
Kolekar et al. [76]	Web services	Medical AI services.
Susilo et al. [51]		Pharmacokinetics modeling.
Peterson et al. [77]		TumorScope Predict platform.
Chaudhuri et al. [49]	Data processing software	Bayesian calibration models.
Moztarzadeh et al. [78]	Cloud infrastructure	ML-based diagnostic models.
Chang et al. [79]	CBCT data integration	Adaptive proton therapy.
Sharma et al. [80]	AI-based middleware	Python Libraries (TensorFlow, Keras, Pandas, and Numpy), CervixNet.
Christenson et al. [81]	MRI calibration systems	MATLAB
Joslyn et al. [82]	Pharmacokinetics software	QSP modeling
Kolokotroni et al. [83]	Biomechanical simulators	Cellular kinetics simulators

HPC—high-performance computing system; QSP—quantitative systems pharmacology; * Multiphysics for computational fluid dynamic (CFD) simulations and a finite element method (FEM) software; ** DCGAN—deep convolutional generative adversarial network; ^#^ Multiphysics for solving the diffusion process of fentanyl through the skin and pharmacokinetics/pharmacodynamics modeling.

**Table 9:** Technical characteristics of the proposed solution: type of data flow, key technologies, analytical methods, and credibility.

Reference	Data Flow	Key Technologies and Analytical Methods	Credibility
Zhang et al. [57]	Bidirectional	DL using CNNs and Bi-LSTM with attention mechanisms (for vulnerability detection in software).	Partial evidence
Meraghni et al. [58]	Bidirectional	ML, AI (data analysis).	Partial evidence
Ahmadian et al. [59]	Bidirectional	DCGAN for reconstructing bone microstructures, CFD for simulating the cement injection, and FEM for analyzing the mechanical stability of the vertebra.	Partial evidence
Ahmadian et al. [60]	Unidirectional	DCGAN to reconstruct the trabecular bone microstructure and FEM to simulate the vertebral fracture response.	Partial evidence
Qi and Cao [61]	Bidirectional	AI-based techniques handle and analyze large datasets, although they do not provide details about specific AI models.	Partial evidence
Lin et al. [62]	Unidirectional	Integration of well-established mathematical models and real-time patient data.	Complete evidence
Bahrami et al. [50]	Bidirectional	Monte Carlo simulations and Markov modeling methods.	Complete evidence
Yankeelov et al. [63]	Bidirectional	Biology-based mathematical modeling, optimal control theory for optimizing treatment protocols, and data assimilation for continuously updating.	Complete evidence
Gamage et al. [64]	Bidirectional	ML for data processing and decision support; and computational physiology models for simulating organ functions and disease processes; FAIR principles (data management).	Complete evidence
Zhu et al. [65]	Unidirectional	Deep learning (U-Net architecture) for image reconstruction; biomechanical modeling of lung motion; and electrical field modeling for simulating lung conductivity.	Sufficient credibility
Bahrami et al. [66]	Unidirectional	Physics-based modeling; Monte Carlo simulations for generating virtual patient populations; and COMSOL Multiphysics for solving the equations governing drug distribution and effects. Markov chain Monte Carlo for creating virtual populations.	Partial evidence
Bahrami et al. [67]	Unidirectional	Physics-based modeling. DT also integrates models for different skin layers and anatomical sites.	Partial evidence
Mosch et al. [68]	Bidirectional	Immunotherapy modeling, optimization algorithms.	Partial evidence
Servin et al. [69]	Unidirectional	Microwave ablation, finite element modeling.	Complete evidence
Pérez-García et al. [70]	Bidirectional	Mathematical chemotherapy models.	Partial evidence
Tai et al. [71]	Bidirectional	GAN and IoMT models.	Sufficient credibility
Jamshidi et al. [72]	Bidirectional	Metaverse and AI integration, Decision Trees, and ML models.	Partial evidence
Kim et al. [73]	Bidirectional	ML, predictive modeling and Python, TensorFlow, Keras.	Partial evidence
Meng et al. [74]	Bidirectional	Causal inference with ML.	Sufficient credibility
Raja et al. [75]	Bidirectional	Random forest analysis.	Partial evidence
Kolekar et al. [76]	Bidirectional	AI.	Sufficient credibility
Susilo et al. [51]	Unidirectional	QSP modeling.	Sufficient credibility
Peterson et al. [77]	Bidirectional	Biophysical simulations.	Partial evidence
Chaudhuri et al. [49]	Bidirectional	Bayesian models, Bayesian optimization.	Sufficient credibility
Moztarzadeh et al. [78]	Bidirectional	AI, IoMT, metaverse, and AI and ML-based classification.	Partial evidence
Chang et al. [79]	Bidirectional	CBCT imaging.	Partial evidence
Sharma et al. [80]	Bidirectional	AI, ML, and simulation models.	Partial evidence
Christenson et al. [81]	Bidirectional	Proper orthogonal decomposition. Simulation models, ML, real-time data analytic.	Sufficient credibility
Joslyn et al. [82]	Unidirectional	QSP pharmacokinetics modeling.	Sufficient credibility
Kolokotroni et al. [83]	Bidirectional	Hypermodeling and cellular modeling.	Sufficient credibility

AI—artificial intelligence; ML—machine learning; QSP—quantitative systems pharmacology; IoMT—Internet of Medical Things; CBCT—cone-beam computed tomography.

## Data Availability

No new data were created or analyzed in this study.
